# Bailcalin Protects against Diabetic Cardiomyopathy through Keap1/Nrf2/AMPK-Mediated Antioxidative and Lipid-Lowering Effects

**DOI:** 10.1155/2019/3206542

**Published:** 2019-07-01

**Authors:** Ran Li, Yuan Liu, Ying-guang Shan, Lu Gao, Fang Wang, Chun-guang Qiu

**Affiliations:** ^1^Department of Cardiology, The First Affiliated Hospital of Zhengzhou University, China; ^2^Department of Endocrinology, The First Affiliated Hospital of Zhengzhou University, China

## Abstract

Previous studies demonstrated that Bailcalin (BAI) prevented cardiac injuries under different disease models. Whether BAI protected against type 2 diabetes mellitus- (T2DM-) associated cardiomyopathy was investigated in this study. T2DM was established by the combination of streptozotocin injection and high-fat diet in mice. BAI was administered daily for 6 months. After evaluating cardiac functions, mice hearts were removed and processed for morphological, biochemical, and molecular mechanism analyses. Neonatal rat cardiomyocytes (NRCM) were isolated and treated with high glucose and palmitate (HG/Pal) for *in vitro* investigation. BAI significantly ameliorated T2DM-induced cardiomyocyte hypertrophy, interstitial fibrosis, and lipid accumulation accompanied by markedly improved cardiac functions in diabetic mice. Mechanically, BAI restored decreased phosphorylation of AMPK and enhanced expression and nuclei translocation of Nrf2. In *in vitro* experiments, BAI also prevented NRCM from HG/Pal-induced apoptosis and oxidative stress injuries by increasing p-AMPK and Nrf2 accumulation. The means by which BAI restored p-AMPK seemed to be related to the antioxidative effects of Nrf2 after silencing AMPK or Nrf2 in NRCM. Furthermore, BAI regulated Nrf2 by inhibiting Nrf2 ubiquitination and consequent degradation mediated by Keap1. This study showed that BAI alleviated diabetes-associated cardiac dysfunction and cardiomyocyte injuries *in vivo* and *in vitro* via Keap1/Nrf2/AMPK-mediated antioxidation and lipid-lowering effects. BAI might be a potential adjuvant drug for diabetes cardiomyopathy treatment.

## 1. Introduction

A pooled analysis of 4.4 million participants showed that the global prevalence of diabetes has quadrupled since 1980 [1, 2]. About 8% of the people worldwide have been diagnosed with diabetes [1]. The cardiovascular system has been demonstrated to be one of the most suffering systems [1]. Diabetes could offset any decrease in cardiovascular morbidity and mortality obtained from the control of other cardiovascular risk factors [1, 2]. Diabetic cardiomyopathy (DCM) has been accepted as one of the most common and severe complications. DCM was characterized with the structure and function impairments of the myocardium, which were independent of hypertension- or coronary artery disease-induced cardiomyopathy. Currently, no effective treatments have been used for preventing DCM, and even some traditional treatments have been demonstrated to have adverse effects for DCM therapy. However, increasing investigations have suggested that natural products, derived from green vegetables, fruits, teas, and daily foods, might be potential drugs for treatment or adjuvant treatment of DCM.

Many underlying mechanisms have involved in the development and progress of DCM [3]. Oxidative stress and lipid toxicity are two main pathogenesis of DCM induced by T2DM [4]. The redox dynamic equilibrium state is disturbed in the progress of diabetes because of decreased antioxidant and increased oxidant in cardiomyocytes, which causes the accumulation of reactive oxygen species (ROS) [4]. Excessively accumulated ROS may react quickly and nonspecifically with proteins, lipids, and DNA, resulting in DNA damage, metabolism aberration, and cell apoptosis [4]. Nuclear factor (erythroid-derived 2)-like 2 (Nrf2) is a cytoprotective transcription factor that controls the expression of many antioxidant factors [5]. Under unstressed normal conditions, Nrf2 is arrested in cytoplasm for quick ubiquitin degradation by binding with an E3 ubiquitin ligase substrate adaptor (Kelch-like ECH-associated protein 1 (Keap1)) [5]. Keap1 possesses several highly reactive cysteines, upon modification by sensing oxidants and electrophiles, which could prevent Keap1-mediated Nrf2 proteasomal degradation, thereby resulting in nuclear accumulation of Nrf2. Nrf2 binds to antioxidant response elements (AREs) in the nucleus to activate the transcription of many antioxidant genes [5]. Activation of Nrf2 has been demonstrated to be beneficial for several cardiovascular disorders including hypertension, heart failure, and diabetic cardiomyopathy. The expression of Nrf2 was significantly downregulated at the late stage of DCM. Studies have demonstrated that restoring the expression and nucleus translocation of Nrf2 could prevent cardiomyocyte from diabetes-associated injuries *in vitro and in vivo* [6]. Lipid toxicity, another important pathological factor in DCM, is caused by dysregulation of enzymes and signaling pathways involved in triacylglycerol, phospholipid, and sphingolipid metabolisms [7]. Adenosine monophosphate-activated protein kinase (AMPK) is a key enzyme in regulating lipid metabolism and utilization [7]. Activated AMPK could promote glycolysis and fatty acid oxidation, autophagy initiation, and glycogen, lipid, and protein syntheses; however, the activity of AMPK was significantly downregulated in DCM. Genetic or pharmaceutic activation of AMPK could effectively decrease lipid accumulation and improve cardiac functions in DCM. Therefore, simultaneous pharmacological regulation of Nrf2 and AMPK might be a beneficial strategy in preventing DCM.

Baicalin (BAI) is a flavonoid component extracted from the traditional Chinese medicine *S. baicalensis*. BAI was shown to inhibit oxidative stress by reinforcing the endogenous antioxidant system through regulating the nuclear translocation of Nrf2 and HO-1 expressions in the alcoholic liver mouse model [8]. In a cerebral ischemia/reperfusion (I/R) mouse model, BAI ameliorated hyperglycemia-exacerbated I/R injuries through regulating mitochondrial functions in an AMPK*α*1-dependent manner [9]. More importantly, BAI has been demonstrated to decrease blood lipids and inflammation [10] in a randomized, double-blind, placebo-controlled trial. In cardiovascular diseases, BAI could prevent embryonic cardiovascular malformation by suppressing the excessive production of ROS and autophagy [11]. BAI has been shown to protect against pressure overload-induced cardiac remodeling through regulating AMPK [12] or PPARs [13]. So far, the role and underlying mechanism of BAI in T2DM-associated DCM have not been reported. Herein, we used an experimental T2DM mouse model to clarify the above formulated issues.

## 2. Materials and Methods

### 2.1. Materials

BAI was purchased from Shanghai Winberb Medical S&T Development Co. Ltd. (Shanghai, China). BAI was provided by the manufacturer with a purity of >99%, which was determined by high-performance liquid chromatography. MG132 was purchased from Beyotime (S1748).

### 2.2. Animals

Male C57BL/6J mice (age: 9-11 weeks, weight: 24.5-25.5 g) were purchased from the Institute of Laboratory Animal Science, Chinese Academy of Medical Science (Beijing, China). All mice were housed for adapting to environment for one week before establishing the diabetic mouse model. All mice have free access to food and water with a 12 h light/dark cycle. All animal experiments abided by the laboratory animal guidelines published by the United States National Institutes of Health (NIH Publication, revised 2011) and were approved by the Animal Care and Use Committee of Zhengzhou University.

### 2.3. Diabetic Mouse Model and BAI Treatment

The type 2 diabetes model was established according to the method used in the previously published literature [14, 15]. Briefly, mice were fed with high-fat diet (HFD) to induce insulin resistance or normal diet (ND) for 3 months. Type 2 diabetes was induced in these insulin-resistant mice with intraperitoneal injection of streptozotocin (STZ) dissolved in 0.1 M citrate buffer pH 4.5 at the dose of 100 mg/kg [14, 15]. Blood glucose was examined, and mice with blood sugar > 250 mg/dL were defined with diabetes and used for following experiments.

Four groups were included in this study: ND with vehicle treatment group (VEH, *n* = 12), ND with BAI treatment group (BAI, *n* = 12), HFD with vehicle treatment after STZ injection group (DM, *n* = 16), and HFD with BAI treatment after STZ injection group (DM/BAI, *n* = 16). BAI was administered to mice by a gastric tube for 4 months. BAI was administered at a dosage of 100 mg/kg/d for every day at 2 pm in the BAI and DM/BAI groups. The dose of BAI was chosen according to previous studies [14, 15]. The VEH and DM groups received the same volume of vehicle for 4 months.

### 2.4. Isolation and Treatment of Cardiomyocytes

#### 2.4.1. Isolation of Cardiomyocytes

Neonatal rat cardiomyocytes (NRCM) were isolated according to a published protocol [16, 17]. Briefly, neonatal rats (0-3-day-old Sprague-Dawley rats) were anesthetized with carbon dioxide and sacrificed by cervical dislocation. Rat hearts were removed and minced into pieces of about 1 mm^3^. Minced cardiac tissues were transferred to a glass bottle and resuspended in trypsin-collagenase 2 buffer. Then, the glass bottle was put into a preheated cell vibrator for 30 min with 100 rpm at 37°C. The cell suspensions were collected into a 50 mL centrifuge tube. Enzyme activity was terminated by addition of horse serum. Fresh trypsin-collagenase 2 buffer was added into the remaining tissue pieces for cellular digestion for another 30 min. Repeated cellular digestion was performed until completely dissociating tissue fragments. Collected cell suspensions were pooled, filtered, centrifuged for 5 min at 1000 rpm, and then resuspended in 12 mL cardiomyocyte culture medium. Cardiomyocytes were isolated from cardiac fibroblasts through delayed adherent culture for 1 hour in a precoated culture dish. After 1 hour of incubation in a humidified incubator at 37°C and 5% CO_2_, cardiac fibroblasts adhered to the culture dish while cardiomyocytes remained to suspend in culture medium. Cardiomyocytes were seeded at a density of 2 × 10^5^/mL for following experiments.

#### 2.4.2. siRNA Transfection

To knock down Nrf2 and AMPK*α*, respectively, Nrf2 siRNA (sc-156128), AMPK*α*2 siRNA (sc-155985), and control siRNA (sc-37007) were purchased from Santa Cruz Biotechnology. siRNAs were transfected into NRCM for 18-24 h by using lipofectamine 200 (Invitrogen). Transfection efficiency was evaluated by Western blot for Nrf2 and AMPK protein expression quantifications, respectively.

#### 2.4.3. Treatment of NRCM

NRCM were treated with high glucose and palmitate (HG/Pal) to mimic T2DM-associated NRCM injuries according to published protocol [18]. Briefly, NRCM were cultured in medium containing D-glucose with a final concentration of 33 mM and palmitate with a final concentration of 200 *μ*M, which could not cause cellular apoptosis in a period of 36 hours in experiment (data not shown). After testing the effects of BAI at different concentrations in NRCM, a final concentration of 20 *μ*M was used for following experiments.

### 2.5. Cardiac Function Evaluation by Echocardiography and Pressure-Volume Loop

Transthoracic echocardiography (Echo) was performed to evaluate mouse cardiac functions by a high-resolution Echo system (Esaote S.P.A., Genoa, Italy) for small animals, equipped with a 10 MHz microprobe. The cardiac functions of mice were assessed under condition of anesthesia with 1.5% isoflurane. The left ventricle (LV) dimension was acquired at a parasternal short axis view. The heart rate (HR), LV end-diastolic diameter (LVEDd), LV end-systolic diameter (LVEDs), LV end-diastolic volume (LVVd), LV end-systolic volume (LVVs), LV ejection fraction (EF), and LV fractional shortening (FS) were calculated by system software. Pressure-volume analysis was performed according to published protocol [19]. After isolating the mouse carotid artery, a 1.4 French Millar catheter transducer was inserted into mouse LV under conditions of anesthesia with 1.5% isoflurane. The PVAN data analysis software was used for data analysis.

### 2.6. Examination of Pathological Morphology Changes in Mice Hearts

Mice hearts were harvested and fixed in 10% formalin for 12-24 h at room temperature. After dehydration, mice hearts were embedded in paraffin for cutting into 4-5 *μ*m thick slices. The slices were stained with hematoxylin and eosin (HE) for examining general morphology and cardiomyocyte surface area (CSA). To observe cardiac fibrosis, the slices were subjected to Masson trichrome staining. The collagen deposition was shown in blue areas. Oil Red staining was performed to examine lipid accumulation in mouse heart tissue. Mice hearts were harvested freshly and embedded in optimal cutting temperature (OCT) medium for frozen sections at a thickness of 8-10 *μ*m. The frozen sections were fixed in 10% formalin for 5 minutes at room temperature and then stained with the Oil Red O reagent for 1 h. After washing with 10% isopropanol, the frozen sections were counterstained with hematoxylin for 30 seconds. All of these stained slices were photographed by the Nikon microscope. The Image-Pro Plus 6.0 software was used to analyze the CSA in HE staining and collagen content in PSR staining.

### 2.7. Western Blot Assay

Snap frozen heart tissue was homogenized, or cardiomyocytes were sonicated in RIPA buffer. Total proteins were extracted and separated by 8%, 10%, or 12% SDS-PAGE gels (according to molecular weight) and then transferred to a nitrocellulose membrane, which was blocked with 5% FBS for 1 h at room temperature and then incubated with primary antibodies overnight at 4°C. The primary antibodies used in this study are ANP (CST, 1 : 500), BNP (CST, 1 : 500), *β*-MHC (CST, 1 : 500), p-AMPK*α* (CST, 1 : 1000), T-AMPK*α* (CST, 1 : 1000), p-ACC (CST, 1 : 500), T-ACC (CST, 1 : 500), c-caspase 3 (CST, 1 : 500), BAX (CST, 1 : 1000), *β*-actin (CST, 1 : 500), Nrf2 (ABCAM, 1 : 500), HO-1 (ABCAM, 1 : 500), CPT-1 (ABCAM, 1 : 500), PGC1-*α* (ABCAM, 1 : 500), 3-NT (ABCAM, 1 : 500), 4-HNE (ABCAM, 1 : 500), Keap1 (ABCAM, 1 : 200), and ubiquitin (ABCAM, 1 : 500). On the next day, blots were washed with Tris-buffered saline containing 0.05% Tween 20 and then incubated with horseradish peroxidase- (HRP-) conjugated secondary antibody for 1 h at room temperature. The enhanced chemiluminescence kit (Thermo) was used to visualize corresponding blots. Image Lab 3.0 was used for blot analysis.

### 2.8. Isolation of RNA for Real-Time Quantitative Polymerase Chain Reaction (RT-qPCR)

Total RNA was isolated using the TRIzol Reagent (Invitrogen, CA, USA) or NRCM according to manufacturer's instruction. The NanoDrop ND-1000 spectrophotometer was used to determine RNA concentrations and purities. The first strand complimentary DNA (cDNA) synthesis kit was purchased for reverse transcription of RNA according to manufacturer's instruction. 2 *μ*g total RNA was reversely transcribed into cDNA. RT-qPCR was performed in a 20 *μ*L reaction buffer, including 10 *μ*L SYBR green dye, 1 *μ*L primers, 1 *μ*L templet, and 8 *μ*L H_2_O, in the Roche RT-qPCR system. Primers used in this study were listed in Table 1. Fluorescence intensity was measured to monitor the amplification of target genes. Comparative cycle time (CT) was used to assess fold changes among different groups.

### 2.9. Nuclei Extraction

To investigate nuclei translocation of Nrf2, the nuclei isolation kit (Nuc-201, Sigma) was purchased to extract nuclei from cardiomyocytes both ex vivo and *in vitro* according to the manufacturer protocol. Briefly, 50 mg cardiac tissue of each sample was homogenized, or 1 × 10^7^ cardiomyocytes were sonicated in 300 mL cold lysis buffer, and then, 600 mL cold 1.8 M Cushion Solution was added. Finally, the mixture was transferred into a new tube preloaded with Sucrose Cushion Solution. Tubes were centrifuged at 30000 g for 30 min. Nuclei were deposited at the bottom of tubes, and the cytoplasmic component existed in the supernatant layer. The supernatant and sediments were used for protein extraction and Western blot analysis.

### 2.10. Coimmunoprecipitation and Ubiquitination Analysis

Protein A+G Agarose was purchased from Beyotime Biotechnology (China, P2012) and was preserved at 4°C. The protein A+G Agarose was upside down for sufficient suspension before use. The culture medium was discarded, and cells were washed 3 times, and then, 1 mL RIPA lysate was added into each dish to lyse cells completely. To avoid nonspecific binding, normal rabbit IgG (1 *μ*g) and protein A+G (20 *μ*L) were added into each sample (containing 1 mg protein) for incubation 2 h at 4°C on a rotary platform, and then, the supernatant solution was collected for coimmunoprecipitation after 2500 rpm centrifugation for 5 minutes. 1 *μ*g of primary antibody was added into each sample for overnight at 4°C on a rotary platform. Protein A+G (40 *μ*L) was added into each sample for incubation for 3 hours on a rotary platform at 4°C. The supernatant solution was discarded after 2500 rpm centrifugation for 5 minutes; the precipitate was washed with PBS with 2500 rpm centrifugation for 5 min × 5 times, and then, 40 *μ*L 1X SDS-PAGE electrophoretic sample buffer was added for resuspension of the precipitate. The collected protein lysate was boiled at 100°C for 10 min and then were loaded on 10% SDS-PAGE for Western blot and ubiquitin analyses. In this study, we isolated Nrf2 first by immunoprecipitation and then detected the ubiquitin by using anti-Ub antibody.

### 2.11. Measurement of MDA, GSH, GSSG, and SOD

50 mg heart tissue of each sample was homogenized in cold lysis buffer according to the manufacturer's protocol. Total proteins were collected by centrifugation at 12000 rpm for 15 min at 4°C. The CBA protein quantitative kit (Thermo) was used to determinate the protein concentration. The lipid peroxidation malondialdehyde (MDA) assay kit (S0131), GSH and GSSG Assay Kit (S0053), and total superoxide dismutase (SOD) assay kit (S0101) with WST-8 were purchased from Beyotime Co. (Shanghai, China). The examinations were performed according to the manufacturer's instructions.

### 2.12. Statistical Analysis

Data were presented as means ± SEM. Comparisons among different groups were performed by one ANOVA, followed by the LSD test using SPSS 19.0. If heterogeneity of variance presented, ANOVA was performed after logarithmic transformation of the data. Statistical significance was registered as *p* < 0.05.

## 3. Results

### 3.1. BAI Prevented Diabetes-Induced Cardiac Remodeling

**Figure 1:**
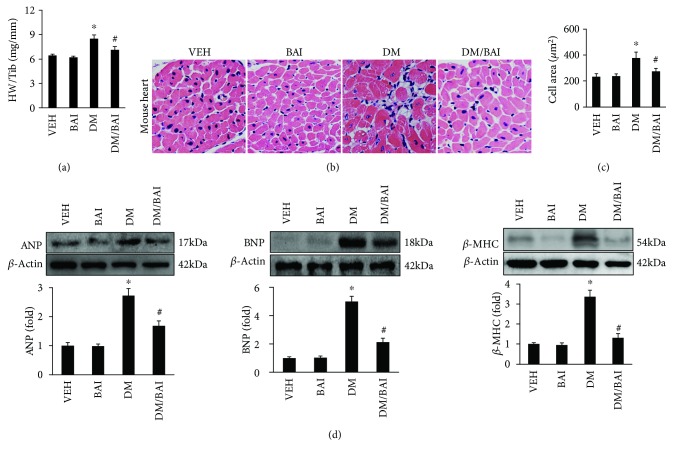
BAI attenuated T2DM-induced cardiac remodeling: (a) heart weight (HW)/tibia length (Tib), (b) HE staining of the left ventricle, (c) calculated cardiomyocyte surface area (CSA), and (d) the expression of cardiac remodeling-associated biomarkers, including ANP, BNP, and *β*-MHC. *N* = 6/group. T2DM: type 2 diabetes mellitus; VEH: vehicle; DM: diabetes mellitus; BAI: Bailcalin. ^∗^ *p* < 0.05 compared with the VEH or BAI group; ^#^ *p* < 0.05 compared with the DM group.

After evaluating cardiac functions with Echo and pressure-volume loops, body weight and heart and lung weights (HW and LW), as well as tibia length (Tib), were recorded. As shown in Figure 1(a), the HW/Tib ratio was significantly increased in diabetic mice compared with nondiabetic mice. HE staining presented cardiomyocyte hypertrophy and inflammatory cell infiltration (Figures 1(b) and 1(c)). BAI treatment effectively decreased the HW/Tib ratio and prevented cardiomyocyte hypertrophy and inflammatory cell infiltration induced by diabetes (Figures 1(a)–1(c)). The HW, LVW, and LVW/Tib were also significantly increased in the DM group compared to the VEH or BAI group; BAI attenuated diabetes-induced increase of HW, LVW, and LVW/Tib. Cardiac remodeling-associated biomarkers, including ANP, BNP, and *β*-MHC, were significantly upregulated in diabetic mice hearts (Figure 1(d)), which were attenuated by BAI (Figure 1(d)). The blood glucose (BG) or triglyceride (TG) levels were significantly increased in the DM or DM/BAI group compared to VEH or BAI (Figure S1, A-D). No difference existed between the DM and DM/BAI groups at the beginning of the experiment (Figure S1, A and C); however, BAI treatment significantly decreased BG and TG in the DM/BAI group compared to the DM group at the end of this experiment (Figure S1, B and D). Although BAI treatment reduced BG and TG significantly after 6 months of treatment, the BG and TG in the DM/BAI group was far more than that in the VEH or BAI group (Figure S1, B). Therefore, the protective effects of BAI in DCM might rely on other regulatory mechanisms.

### 3.2. BAI Alleviated Diabetes-Induced Myocardial Fibrosis

**Figure 2:**
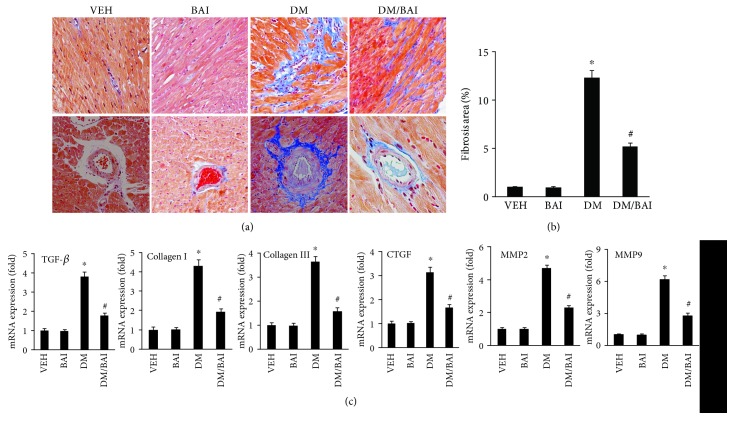
BAI alleviated T2DM-induced cardiac fibrosis: (a) Masson staining for cardiac fibrosis in the mouse left ventricle, (b) the calculated fibrosis area among different groups, and (c) the mRNA expression of fibrosis-associated biomarkers, including TGF-*β*, collagen I, collagen III, CTGF, MMP2, and MMP 9. *N* = 6/group. VEH: vehicle; DM: diabetes mellitus; BAI: Bailcalin. ^∗^ *p* < 0.05 compared with the VEH or BAI group; #*p* < 0.05 compared with the DM group.

Masson trichrome staining was applied to assess cardiac fibrosis. As shown in Figure 2, diabetes caused distinctive interstitial and perivascular fibrosis in the heart (Figure 2(a)), which was attenuated by BAI (Figures 2(a) and 2(b)). RT-PCR analysis showed increased expression of profibrotic genes, including TGF-*β*, collagen I, collagen III, CTGF, MMP2, and MMP9, increased in the DM group (Figure 2(c)), and BAI effectively blocked these changes (Figure 2(c)).

### 3.3. BAI Improved Diabetes-Induced Cardiac Dysfunction

**Figure 3:**
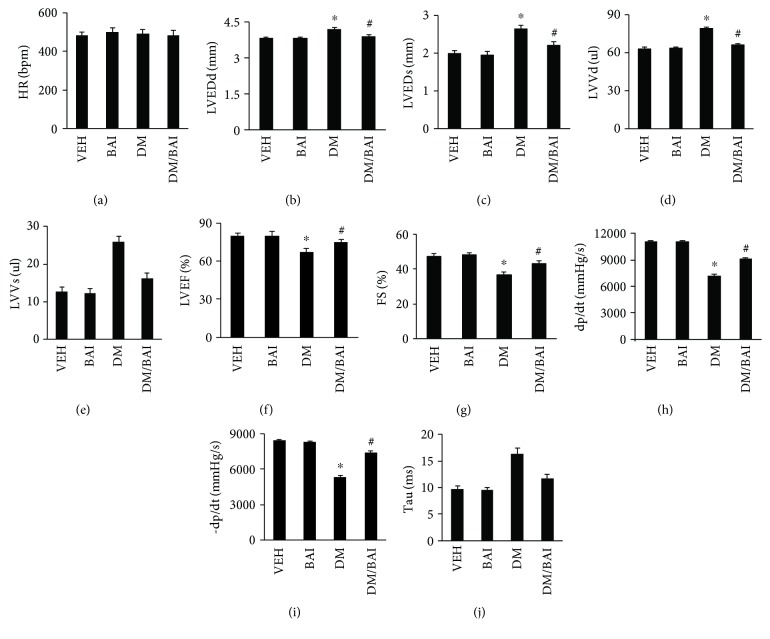
BAI improved T2DM-induced cardiac dysfunction. Parameters of cardiac functions examined by echocardiography: (a) heart rate (HR), (b) left ventricle (LV) end-diastolic diameter (LVEDd), (c) LV end-systolic diameter (LVEDs), (d) LV end-diastolic volume (LVVd), (e) LV end-systolic volume (LVVs), (f) LV ejection fraction (EF), and (g) LV fractional shortening (FS); parameters examined by pressure-volume loop: (h) dp/dt, (i) -dp/dt, and (j) Tau. *N* = 12 in the VEH and BAI groups; *N* = 16 in the DM and DM+BAI groups. VEH: vehicle; DM: diabetes mellitus; BAI: Bailcalin. ^∗^ *p* < 0.05 compared with the VEH or BAI group; #*p* < 0.05 compared with the DM group.

Echocardiography was performed to evaluate cardiac function before sacrificing mice at the end of this experiment. There was no difference of HRs among different groups (Figure 3(a)). The results evidenced that LVEDd, LVEDs, LVVd, and LVVs in the DM group were significantly increased (Figures 3(b)–3(e)), but LVEF and FS (Figures 3(f) and 3(g)) were decreased compared with the nontreated group. However, BAI markedly decreased LVEDd, LVEDs, LVVd, and LVVs and increased LVEF and FS compared with the DM group (Figures 3(b)–3(g)). In pressure-volume loop analysis, DM caused significantly decreased dp/dt and -dp/dt and increased Tau compared to the VEH or BAI group (Figures 3(h)–3(j)); however, BAI improved these changes (Figures 3(h)–3(j)). Thus, BAI could significantly attenuate T2DM-associated mouse cardiac dysfunction.

### 3.4. BAI Improved Lipid Metabolism in the Diabetes Mouse Heart

**Figure 4:**
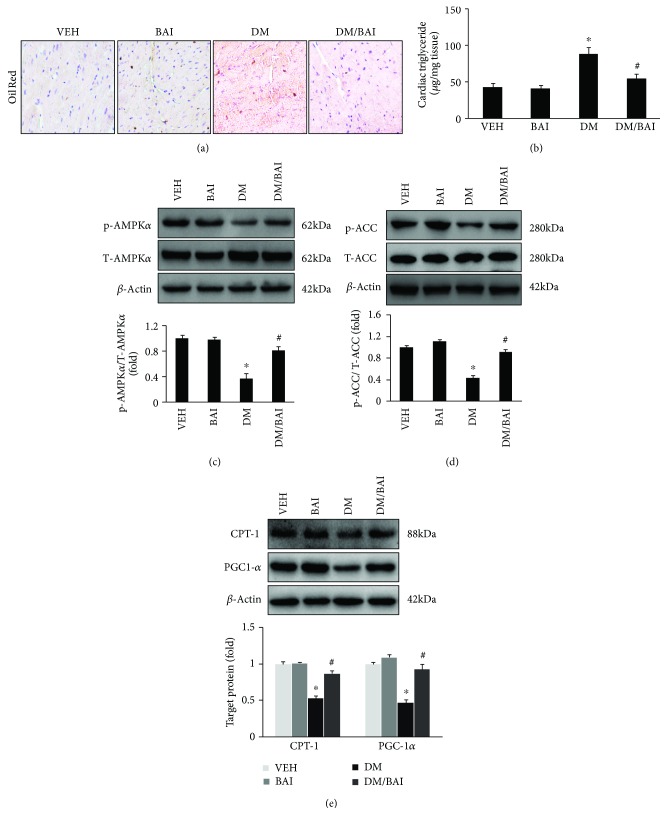
BAI decreased lipid accumulation in the heart of the T2DM mouse: (a) Oil Red staining for examining lipid accumulation, (b) cardiac triglyceride level in the mouse heart, (c) expression of phosphorylated AMPK*α* (p-AMPK*α*) and total AMPK*α* (T-AMPK*α*), (d) expression of p-ACC and T-ACC, and (e) expression of CPT-1 and PGC1-*α*; *β*-actin was selected as internal reference. *N* = 6/group. VEH: vehicle; DM: diabetes mellitus; BAI: Bailcalin. ^∗^ *p* < 0.05 compared with the VEH or BAI group; #*p* < 0.05 compared with the DM group.

Lipotoxicity is more severe in T2DM than in T1DM, which is also a main characteristic in T2DM patients. Therefore, it is meaningful to investigate whether BAI could effectively attenuate lipid accumulation in the T2DM mouse heart. Oil Red staining showed the massive lipid accumulation in the T2DM mouse heart (Figure 4(a)), which could be alleviated by BAI (Figure 4(a)). Furthermore, the level of cardiac triglyceride was significantly increased in the DM mouse heart and was decreased by BAI (Figure 4(b)). Moreover, we found that the phosphorylation of AMPK, a key regulator in cardiac lipid metabolism, was significantly downregulated (Figure 4(c)), and BAI effectively restored AMPK phosphorylation (Figure 4(c)). The phosphorylated AMPK could phosphorylate ACC to blunt its activity. Decreased AMPK was accompanied by decreased ACC phosphorylation but increased accumulation of malonyl-CoA, which caused CPT-1 inhibition. In this study, we also presented that phosphorylation of ACC and expression of CPT-1 were significantly downregulated in the DM mouse heart (Figures 4(d) and 4(e)), and BAI restored ACC phosphorylation and CPT-1 expression (Figures 4(d) and 4(e)). Thus, our investigation suggested that the beneficial effects of BAI on lipid metabolism might be associated with the AMPK-ACC-CPT-1 pathway. In addition, we also examined the expression of PGC1-*α*, a key regulator for fatty acid *β*-oxidation, and the result showed DM-induced downregulation of PGC1-*α*, which could be upregulated by BAI (Figure 4(e)).

### 3.5. BAI Decreased Oxidative Stress in DCM

**Figure 5:**
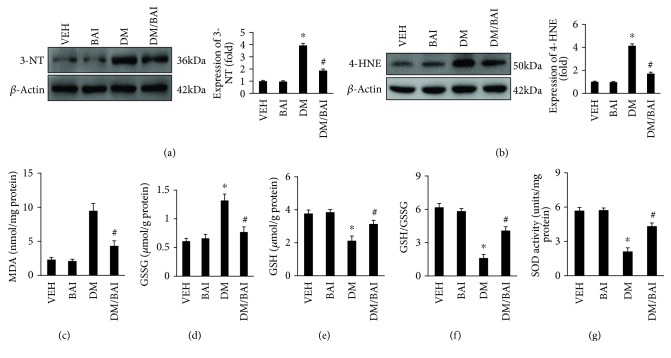
BAI decreased oxidative stress in DCM. Representative blots and calculated expression levels of (a) 3-NT and (b) 4-HNE, examining the (c) MDA, (d) GSSG, (e) GSH, (f) GSH/GSSG ratio, and (g) SOD; *N* = 6/group. VEH: vehicle; DM: diabetes mellitus; BAI: Bailcalin. ^∗^ *p* < 0.05 compared with the VEH or BAI group; #*p* < 0.05 compared with the DM group.

Oxidative stress has been demonstrated to exert a key role in the development of DCM [4]. As shown in Figure 5, the T2DM mouse heart exhibited markedly increased oxidative stress evidenced by increased expression of the nitrosative stress indicator 3-NA and lipid peroxidation indicator 4-HNE (Figures 5(a) and 5(b)); both of which were downregulated by BAI (Figures 5(a) and 5(b)). Lipid peroxidation production and the activity of enzymes related to oxidative stress were investigated. Malondialdehyde (MDA), a key indicator of lipid peroxidation, and GSSG, a key indicator of oxidative stress, were significantly increased in the T2DM mouse heart (Figures 5(c) and 5(d)), but GSH and SOD were decreased (Figures 5(e) and 5(f)). These indicated the destroyed equilibrium between prooxidative stress and antioxidant stress substrates in T2DM. Obviously, BAI restored GSH and SOD but decreased MDA and GSSG (Figures 5(c)–5(g)).

### 3.6. BAI Enhanced Nrf2 Activity

**Figure 6:**
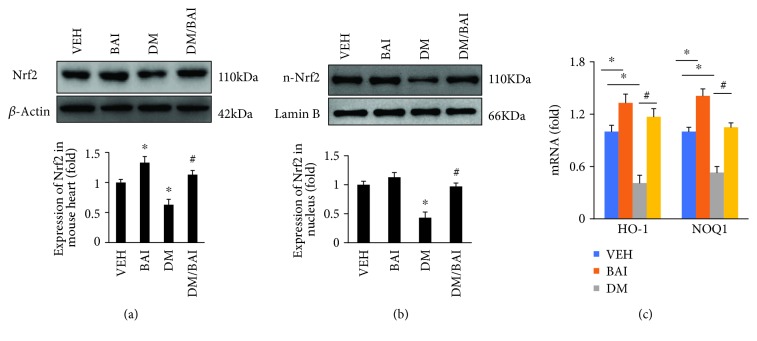
BAI enhanced Nrf2 activity. Representative blots and calculated expression levels of (a) Nrf2 in mouse heart tissue and (b) Nrf2 in the nucleus and the (c) mRNA expression level of HO-1 and NOQ1. *β*-Actin was selected as the internal reference. *N* = 6/group. VEH: vehicle; DM: diabetes mellitus; BAI: Bailcalin. ^∗^ *p* < 0.05 compared with the VEH or BAI group; #*p* < 0.05 compared with the DM group.

Natural compounds of flavonoids have been shown to have potent antioxidative stress function, and one of the underlying mechanisms relates to the regulation of the Nrf2/ARE pathway. Moreover, some previous investigations also indicated that BAI could regulate the activity of Nrf2. In this study, our results showed that expression of Nrf2 was significantly downregulated (Figures 6(a) and 6(b)), and downstream genes of Nrf2, including HO-1 and NQO1 (Figure 6(c)), were also downregulated in the T2DM mouse heart. However, BAI restored the Nrf2 expression, and nucleus translocation resulted in overexpression of HO-1 and NOQ1 (Figure 6(a)–6(c)). Accordingly, activation of the Nrf2 pathway underlies the protection provided by BAI against T2DM-associated oxidative stress injuries in the mouse heart.

### 3.7. BAI Exerted Antioxidative Effects via AMPK Dependent on Nrf2

**Figure 7:**
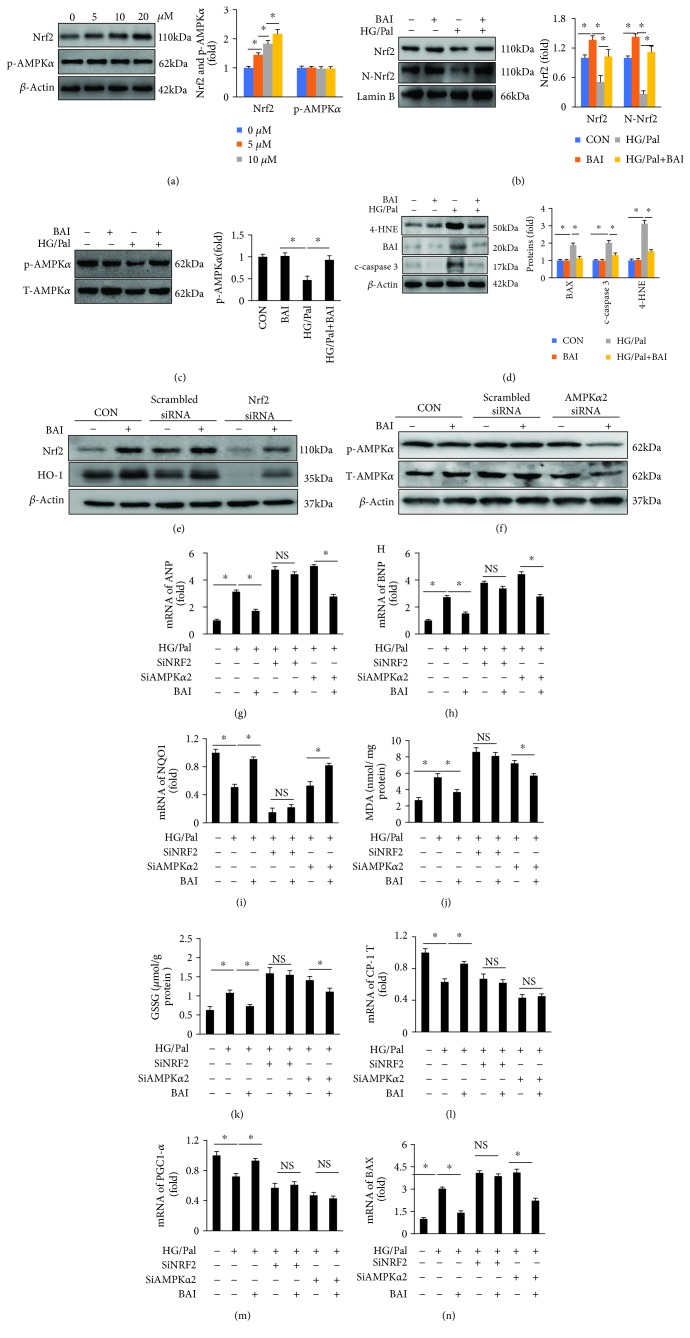
BAI exerted antioxidant effects via AMPK-dependent effects on Nrf2. (a) BAI increased Nrf2 but not p-AMPK*α* in a concentration-dependent manner. (b) BAI restored the expression and nucleus translocation of Nrf2 in HG/Pal-treated NRCM. (c) BAI restored p-AMPK*α* in HG/Pal-treated NRCM. (d) BAI inhibited the expression of 4-HNE, BAX, and cleaved caspase 3 (c-caspase 3) induced by HG/Pal treatment in NRCM. (e) Representative Western blots of Nrf2 and HO-1 expressions with or without Nrf2 silencing; (f) representative blots of p-AMPK*α* and total AMPK*α* (T-AMPK*α*) expressions with or without AMPK*α* silencing; mRNA expressions of (g) ANP, (h) BNP, and (i) NQO1; examination accumulation of (j) MDA and (k) GSSG; mRNA expressions of (l) CPT-1, (m) PGC1-*α*, and (n) BAX. HG: high glucose; Pal: palmitate; BAI: Bailcalin. *β*-Actin was selected as the internal reference. The final concentration of 20 *μ*M BAI was used in experiments; each experiment was repeated three times independently. ^∗^ *p* < 0.05 compared with the indicated group.

NRCM were prepared to investigate the potential BAI mechanism of action *in vitro*. The expression and nuclear translocation of Nrf2 were increased in a concentration-dependent (0, 5, 10, and 20 *μ*M) manner after BAI treatment (Figure 7(a)), while p-AMPK*α* showed no significant change induced by BAI (Figure 7(a)). NRCM were treated with HG/Pal to establish the diabetes-associated cellular model. HG/Pal treatment caused a significant reduction of p-AMPK and Nrf2 (Figures 7(b) and 7(c)), and BAI treatment effectively increased the expression of p-AMPK and Nrf2 (Figures 7(b) and 7(c)). The lipid peroxidation indicator 4-HNE was also significantly accumulated in HG/Pal-treated NRCM, which could be prevented by BAI (Figure 7(d)). Meanwhile, the expression of proapoptosis proteins, including BAX and cleaved caspase 3 (c-caspase 3), was upregulated in NRCM (Figure 7(d)), and BAI could markedly inhibit their expression (Figure 7(d)). These results indicated that BAI could attenuate T2DM-associated cardiomyocyte injuries by enhancing the expression of Nrf2 and p-AMPK *in vitro*.

To investigate the interaction between AMPK and Nrf2, specific siRNAs were used to silence Nrf2 and AMPK, respectively. As shown in Figures 7(e) and 7(f), siRNA transfection successfully inhibited the expression of Nrf2 and AMPK. BAI could not increase the expression of Nrf2 or AMPK after transfection with siRNA for AMPK or Nrf2, respectively, in normal cardiomyocytes (Figures 7(e) and 7(f)). HG/Pal treatment increased expression of ANP and BNP (Figures 7(g) and 7(h)), which were inhibited by BAI (Figures 7(g) and 7(h)). BAI inhibited HG/Pal treatment used for mimicking T2DM effects that induced overexpression of ANP and BNP after AMPK*α* silencing; however, BAI failed to show similar effects after Nrf2 silencing (Figures 7(g) and 7(h)). Nrf2 silencing caused downregulation of HO-1 and NQO1 (Figures 7(e) and 7(i)). BAI treatment could still upregulate NQO1 expression after silence of AMPK*α* (Figure 7(i)). The MDA and GSSG were significantly accumulated in NRCM after HG/Pal treatment (Figures 7(j) and 7(k)), which were blunted by BAI (Figures 7(j) and 7(k)). BAI also prevented HG/Pal-induced increase of MDA and GSSG after silence of AMPK*α* but showed no effect on MDA and GSSG after Nrf2 silencing (Figures 7(j) and 7(k)). BAI could restore CPT-1 and PGC1-*α* reduced by HG/Pal (Figures 7(l) and 7(m)), but this function was eliminated after silence of AMPK and Nrf2, respectively (Figures 7(l) and 7(m)). Finally, BAI treatment could inhibit BAX mRNA expression with or without AMPK*α*2 silencing but could not inhibit BAX mRNA expression after Nrf2 silencing (Figure 7(n)). Through the above descriptions, we could conclude the following.

### 3.8. BAI Prevented Nrf2 Ubiquitination and Modified Keap1

**Figure 8:**
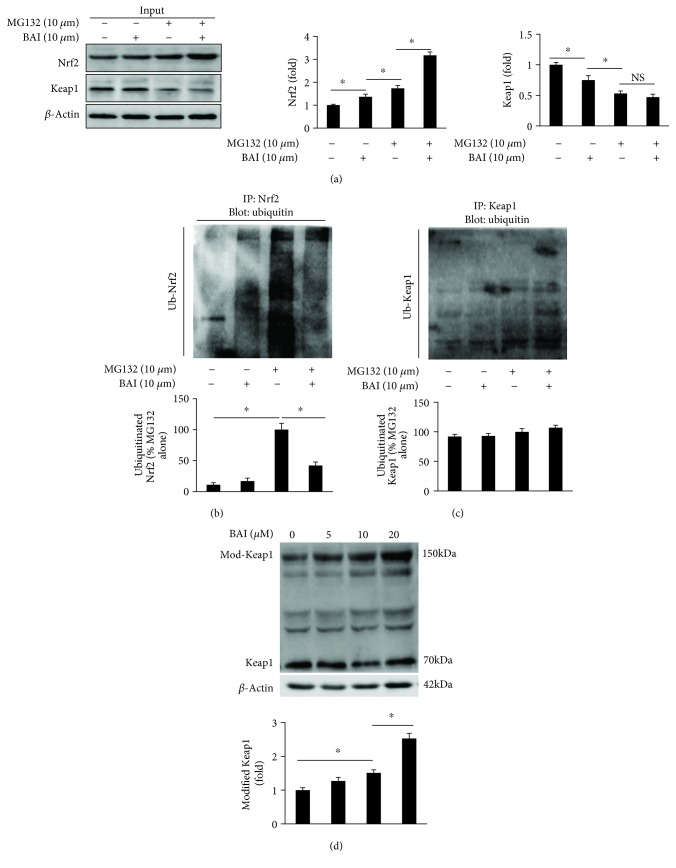
BAI modified Nrf2 ubiquitination: (a) representative blots of Nrf2 and Keap1. (b) Proteins were immunoprecipitated with anti-Nrf2 antibody for ubiquitin detection. (c) Proteins were immunoprecipitated with anti-Keap1 antibody for ubiquitin detection. (d) Detection of modified Keap1 after BAI treatment. NRCM were pretreated with MG132 (10 *μ*M) for 1 h and then incubated with or without BAI (20 *μ*M) for 12 h; each experiment was repeated three times independently. ^∗^ *p* < 0.05 compared with the indicated group.

To further investigate the underlying mechanism for BAI-mediated accumulation of Nrf2 in nuclei, experiments were designed to detect the status of Nrf2 ubiquitination and Keap1 modification after BAI treatment. After treatment of NRCM with MG132, a proteasome-specific inhibitor, or BAI, Nrf2 and Keap1 were immunoprecipitated for examining the ubiquitination of Nrf2 and Keap1, respectively. As presented in Figure 8, Nrf2 expression was upregulated after treatment with MG132 and BAI alone or in combination. But expression of Keap1 was downregulated in different treatment groups compared to the control group (Figure 8(a)). Meanwhile, the ubiquitination of Nrf2 (Figure 8(b)) instead of Keap1 (Figure 8(c)) was significantly reduced in the combination treatment (MG132 and BAI) group. These results definitely showed that enhanced expression of Nrf2 was, at least partly, regulated by inhibiting ubiquitination-mediated Nrf2 degradation; however, the decreased Keap1 expression seemed not to be due to ubiquitination degradation. Previous studies showed that some phytochemicals, such as sulforaphane [20] and quercetin [21], induced the production of modified Keap1, which was about 150 kDa. As shown in Figure 6(d), a blot of about 150 kDa was detected after treatment with BAI. Thus, the downregulation of Keap1 mediated by BAI might be associated with the formation of a modified Keap1 protein complex.

## 4. Discussion

T2DM, composing about 90-95% of the diabetes cases, is the most world spread metabolic disease [2, 3, 22]. Several key pathological abnormalities, including hyperglycemia, hyperlipidemia, insulin resistance, and abnormal insulin secretion because of impaired *β*-cell function, have been demonstrated to be involved in T2DM patients [2, 3, 22]. The T2DM animal models should cover all of the key pathogenic abnormalities listed above. One of the nongenetic models of T2DM used in this study (combined with HFD and STZ injection) has been suggested to mimic most of these metabolic abnormalities observed in human T2DM [14, 15]. In this study, T2DM-induced cardiomyopathy was characterized by cardiac remodeling, morphological abnormality, fibrosis, lipid accumulation, and cardiac dysfunction. But these pathological changes of the heart could be remarkably prevented by BAI treatment. Mechanistically, BAI could effectively increase phosphorylation of AMPK*α* resulting in improved lipid metabolism and decreased lipotoxicity and also promote the accumulation and nuclei translocation of Nrf2 to protect against oxidative stress in T2DM mice hearts. Through silencing of Nrf2 in HG/Pal-treated NRCM, we showed that the regulation of AMPK by BAI might be associated with its function for counteracting oxidative stress in cardiomyocyte. Finally, we also demonstrated that BAI might prevent the combination of Keap1 and Nrf2, resulting in decreased ubiquitination and consequent degradation of Nrf2.

Activation of AMPK plays essential roles in regulating lipid metabolism in a mammalian heart [23]. Phosphorylated AMPK (p-AMPK) could promote fatty acid to enter into the mitochondria for fatty acid oxidation (FAO) via regulation of carnitine palmitoyl CoA transferase 1 (CPT-1), which is the rate-limiting enzyme of FAO in the heart [23]. CPT-1 could be inhibited by malonyl-CoA, which is a direct downregulation target of acetyl-CoA carboxylase (ACC) [23]. Activated AMPK could inhibit the activity of ACC to reduce production of malonyl-CoA resulting in CPT-1 activation [23]. In the progress of diabetic cardiomyopathy, the activity of AMPK and CPT-1 was significantly downregulated, but the activity of ACC was upregulated [18]. It has also been reported that the activity and expression of PGC1-*α* significantly reduced in the late stage of DCM, resulting in aggravated mitochondrial damage [24]. The expression of PGC1-*α* could also be restored after AMPK activation. Consistent with previous studies, we found that phosphorylation of AMPK and expression of CPT-1 and PGC1-*α* were reduced in the T2DM mouse heart and HG/Pal-treated NRCM; however, BAI could effectively reduce these pathologic changes. These results indicated that the protective effects of BAI for attenuating DCM might be partially ascribed to the regulation of the AMPK pathway.

Oxidative stress is another important pathological process in the development and progress of DCM [4]. Although many mechanisms, including inflammation, autophagy, cardiac hypertrophy, and fibrosis, along with cardiomyocyte apoptosis, have been demonstrated to be involved in the pathological remodeling induced by diabetes, cardiac injuries caused by these pathological mechanisms could be significantly improved via strategies targeting oxidative stress [4]. Because few specific antioxidants are effective for DCM treatment, it is necessary to develop chemicals and drugs against oxidative stress during DCM. Nrf2 has been suggested to be a potential target for many chronic diseases, including neurodegenerative diseases, metabolic diseases, and cardiovascular diseases [5]. Nrf2 could induce the expression of a broad panel of antioxidant genes, like heme oxygenase-1 (HO-1), glutathione-S-transferase, NAD(P)H:quinone oxidoreductase- (NQO-) 1, and superoxide dismutase, via binding to antioxidant response elements (AREs) [5, 6]. Nrf2 has also been verified to downregulate in the heart of diabetic animals and patients. In experimental diabetic models, Nrf2 deficiency enhanced oxidative and nitrosative stresses and led to early-stage cardiac injuries and dysfunction [3]. The diabetic model with Nrf2 deficiency displayed exaggerated cardiac fibrosis and increased ROS production. Treatment with Nrf2 inducers, such as sulforaphane and myricetin, could effectively protect mice hearts from diabetes-induced injuries [25, 26]. In this study, we exhibited that BAI could increase the expression of Nrf2, HO-1, SOD, and NQO1. Our results definitely indicate that BAI might be an effective inducer of Nrf2 for protecting against DCM.

Next, we would discuss the underlying relationship between AMPK*α* and Nrf2 with BAI treatment. Some other studies have suggested that hyperphosphorylation of AMPK promoted Nrf2 activation and nuclei translocation [27–29]. Our data demonstrated that BAI could not stimulate the AMPK phosphorylation at the baseline *in vitro* but could activate Nrf2 *in vivo and in vitro* at both baseline and pathological status. These results suggested that BAI might not directly regulate the AMPK activity. After AMPK*α* silencing by siRNA, we further showed that BAI-mediated AMPK*α* phosphorylation might be associated with Nrf2 regulation. The underlying mechanisms for reduced AMPK phosphorylation in DCM were partly ascribed to excessive oxidative stress [30], because excessive oxidative stress contributes to lipid peroxidation resulted in accumulation of 4-HNE [30]. Overproduction of 4-HNE inhibits the activity of LKB1 resulting in decreased AMPK phosphorylation [31]. The present study showed that BAI treatment inhibited 4-HNE accumulation via enhancing Nrf2 activity. A previous study also supported this view that Nrf2 could restore AMPK phosphorylation via reducing 4-HNE accumulation in DCM [15].

Nrf2 was regulated by an E3 ubiquitin ligase complex containing Keap1, Cullin3 (CUL3), and RING-box protein 1 (RBX1) [5]. Under normal conditions, ubiquitinated Nrf2 is degraded by a highly effective proteasomal degradation system, and this constitutive degradation maintains the activity and quantity of Nrf2 at a very low level in variety of cells [5]. The Keap1-GUL3-RBX1 complex will be destroyed when exposed to electrophiles and ROS, which modify the cysteine residues in Keap1, resulting in Nrf2 accumulation in the nucleus and activation of ARE-associated gene transcription [5]. In this study, a significant reduction of Nrf2 ubiquitination, but not Keap1 ubiquitination, was observed *in vitro*. Previous study demonstrated that cysteine residues (C151) in Keap1 were essential for repressing Nrf2 nucleus translocation [32]. Several chemical compounds, including TBHQ [32], sulforaphane [32], and quercetin [21], could induce C151-dependent Keap1 changes which resulted in protection of Nrf2 against Keap1-mediated ubiquitination degradation. According to previous studies, cysteine residue (C151) often participates in reversible disulfide bonds resulting in altered electrophoretic mobility [21, 32]. Previous studies suggested that some compounds, such as TBHQ, sulforaphane, and quercetin, could modify the cysteine residue in Keap1 resulting in an apparent molecular mass about 150 kDa. In this study, we also showed that BAI treatment increased the modified Keap1 in an apparent molecular mass about 150 kDa. Therefore, we concluded here that BAI-induced increase in Nrf2 expression was at least partially due to inhibition of Nrf2 ubiquitination degradation by modifying cysteine residues in Keap1.

Besides this canonical pathway of Keap1-Nrf2 pathway activation, the Akt/GSK-3*β* pathway and EKR signaling have also been demonstrated to be involved in regulating Nrf2 activity and nucleus accumulation [25, 33]. It also has been reported that BAI might be involved in regulation of Akt/GSK-3*β* [34]and ERK [35]signaling pathways; so, some other mechanisms might be also involved in promoting Nrf2 overexpression and nuclei translocation after BAI treatment; more experiments are needed to clarify these potential regulating mechanisms.

## 5. Conclusion

Overall, our study strongly indicates that BAI could effectively alleviate diabetes-associated cardiac injuries. The underlying mechanisms might be associated with restored AMPK activity and enhanced Nrf2 expression. Moreover, BAI was shown to regulate AMPK activity by controlling oxidative stress via activation of Nrf2. BAI might be a valuable adjuvant drug for the treatment of diabetes cardiomyopathy.

## Figures and Tables

**Table 1:** 

Genes	Genus	Forward primer	Reverse primer
TGF-*β*	Mouse	GCTGAACCAAGGAGACGGAA	GGGCTGATCCCGTTGATTTC
Collagen I	Mouse	GTAACGATGGTGCTGTTGGTG	CACCATTGGCACCTTTAGCG
Collagen III	Mouse	CCCTGGTCCACAAGGATTACA	CACCAGAATCACCCTTGCCT
CTGF	Mouse	AGAACTGTGTACGGAGCGTG	GTGCACCATCTTTGGCAGTG
MMP2	Mouse	AACGGTCGGGAATACAGCAG	TGGTAAACAAGGCTTCATGGG
MMP9	Mouse	CAGACGTGGGTCGATTCCAA	CGCGGCAAGTCTTCAGAGTA
ANP	Rat	TTCTCCATCACCAAGGGCTTC	CACCGCACTGTATACGGGATT
BNP	Rat	GAAGGACCAAGGCCTCACAAA	AACTTCAGTGCGTTACAGCC
NOQ1	Rat	CCACGCAGAGAGGACATCAT	TCAGATTCGACCACCTCCCA
BAX	Rat	TTCATCCAGGATCGAGCAGA	AATTCGCCTGAGACACTCGC
CPT-1	Rat	CCTACCACGGCTGGATGTTT	TACAACATGGGCTTCCGACC
PGC1-*α*	Rat	AACTCTCTGGAACTGCAGGC	GCTTTGGCGAAGCCTTGAAA

## Data Availability

Readers are able to access data in this manuscript at any time if they request. If the reader wants to access the data, please email to the first author (Ran Li, email address: fcclir2@zzu.edu.cn) or the corresponding author (Fang Wang, fccwangf4@zzu.edu.cn) at any time.

## Supplementary Materials

Supplementary Materials Supplementary Figure 1: BAI attenuated diabetes-induced cardiac hypertrophy: (A) tibia length (Tib), (B) heart weight (HW), (C) left ventricle weight (LVW), and (D) LVE/Tib. *N* = 12 in the VEH and BAI groups; *N* = 16 in the DM and DM+BAI groups; *p* < 0.05 compared with the VEH or BAI group; ^#^ *p* < 0.05 compared with the DM group. Supplementary Figure 2: BAI reduced blood glucose and plasma triglycerides: (A) the blood glucose (BG) at the beginning of the experiment, (B) the BG at the end of this experiment, (C) the plasma triglycerides (TG) at the beginning of the experiment, and (D) the plasma TG at the end of this experiment. *N* = 12 in the VEH and BAI groups; *N* = 16 in the DM and DM+BAI groups; *p* < 0.05 compared with the VEH or BAI group; ^#^ *p* < 0.05 compared with the DM group.
