# Supplementary figures and images for: Bailcalin Protects against Diabetic Cardiomyopathy through Keap1/Nrf2/AMPK-Mediated Antioxidative and Lipid-Lowering Effects
Source: Oxid Med Cell Longev. 2019 Jul 1;2019:3206542. doi: 10.1155/2019/3206542 (PMC6636513; doi:10.1155/2019/3206542)

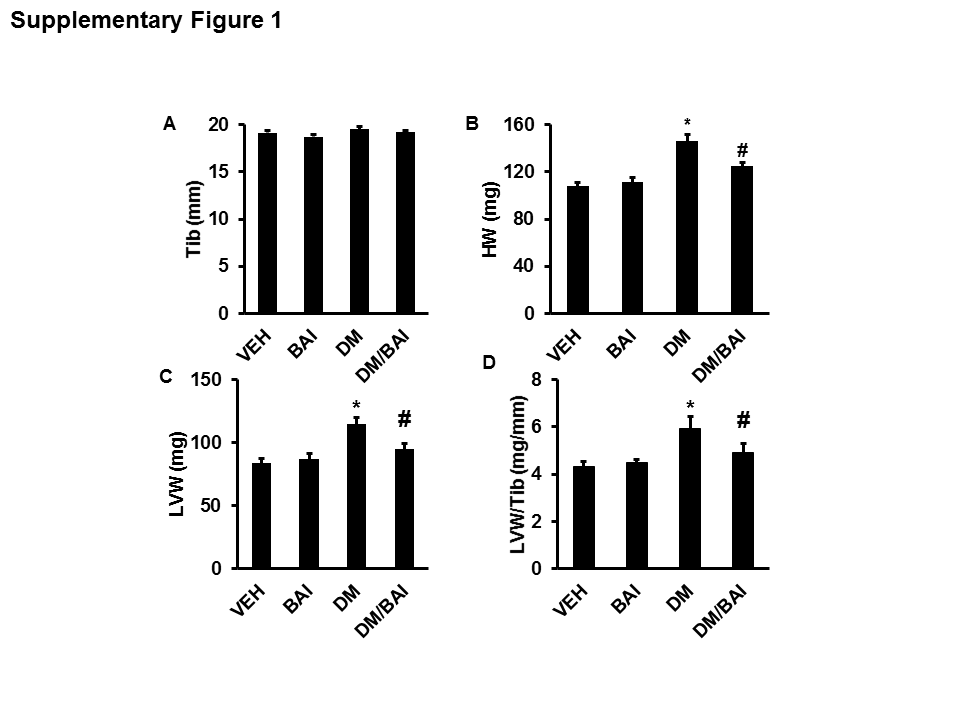


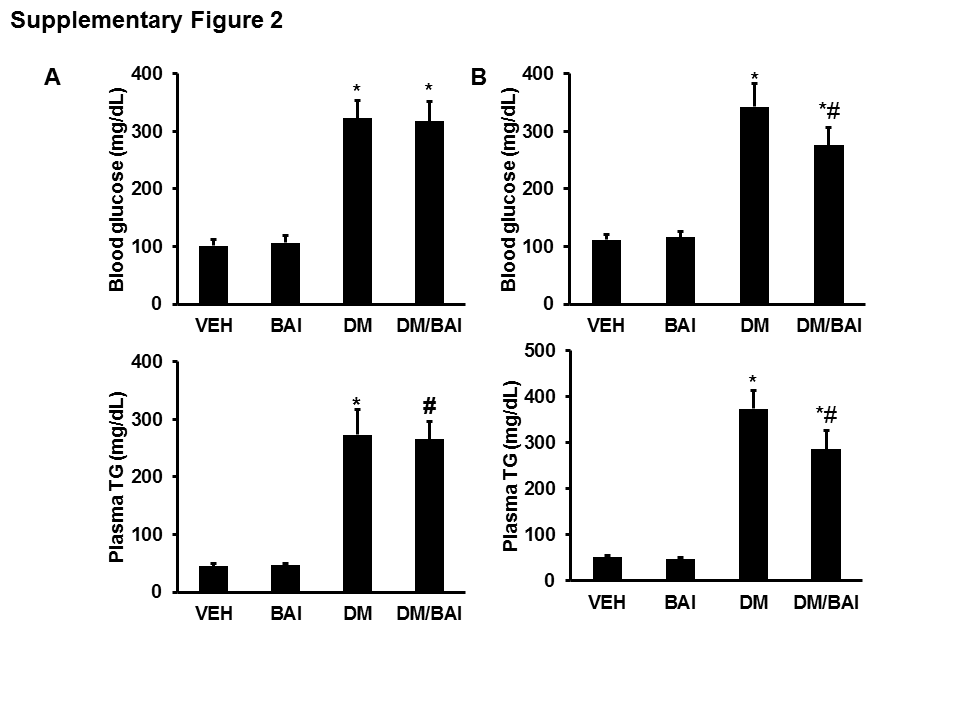

Supplement: Supplementary Materials — Supplementary Figure 1: BAI attenuated diabetes-induced cardiac hypertrophy: (A) tibia length (Tib), (B) heart weight (HW), (C) left ventricle weight (LVW), and (D) LVE/Tib. N = 12 in the VEH and BAI groups; N = 16 in the DM and DM+BAI groups; p < 0.05 compared with the VEH or BAI group; # p < 0.05 compared with the DM group. Supplementary Figure 2: BAI reduced blood glucose and plasma triglycerides: (A) the blood glucose (BG) at the beginning of the experiment, (B) the BG at the end of this experiment, (C) the plasma triglycerides (TG) at the beginning of the experiment, and (D) the plasma TG at the end of this experiment. N = 12 in the VEH and BAI groups; N = 16 in the DM and DM+BAI groups; p < 0.05 compared with the VEH or BAI group; # p < 0.05 compared with the DM group. [file 3206542.f1.docx]
